# Inversions and adaptation to the plant toxin ouabain shape DNA sequence variation within and between chromosomal inversions of *Drosophila subobscura*.

**DOI:** 10.1038/srep23754

**Published:** 2016-03-31

**Authors:** Cinta Pegueroles, Albert Ferrés-Coy, Maria Martí-Solano, Charles F Aquadro, Marta Pascual, Francesc Mestres

**Affiliations:** 1 Departament de Genètica and IRBio, Facultat de Biologia, Universitat de Barcelona, Barcelona 08028, Spain; 2 Centre for Genomic Regulation (CRG), The Barcelona Institute of Science and Technology, Dr. Aiguader 88, Barcelona 08003, Spain; 3 Universitat Pompeu Fabra (UPF), Barcelona, Spain; 4 Department of Neurochemistry and Neuropharmacology, IIBB-CSIC, Barcelona, Spain; 5 Research Programme on Biomedical Informatics (GRIB), Department of Experimental and Health Sciences, Universitat Pompeu Fabra, IMIM (Hospital del Mar Medical Research Institute), Dr. Aiguader, 88, 08003 Barcelona, Spain; 6 Department of Molecular Biology and Genetics, Cornell University, Ithaca, New York 14853, USA

## Abstract

Adaptation is defined as an evolutionary process allowing organisms to succeed in certain habitats or conditions. Chromosomal inversions have the potential to be key in the adaptation processes, since they can contribute to the maintenance of favoured combinations of adaptive alleles through reduced recombination between individuals carrying different inversions. We have analysed six genes (*Pif1A, Abi, Sqd, Yrt, Atpα* and *Fmr1*), located inside and outside three inversions of the O chromosome in European populations of *Drosophila subobscura*. Genetic differentiation was significant between inversions despite extensive recombination inside inverted regions, irrespective of gene distance to the inversion breakpoints. Surprisingly, the highest level of genetic differentiation between arrangements was found for the *Atpα* gene, which is located outside the O_1_ and O_7_ inversions. Two derived unrelated arrangements (O_3+4+1_ and O_3+4+7_) are nearly fixed for several amino acid substitutions at the *Atpα* gene that have been described to confer resistance in other species to the cardenolide ouabain, a plant toxin capable of blocking ATPases. Similarities in the *Atpα* variants, conferring ouabain resistance in both arrangements, may be the result of convergent substitution and be favoured in response to selective pressures presumably related to the presence of plants containing ouabain in the geographic locations where both inversions are present.

Adaptation is a major evolutionary mechanism that allows organisms to live in certain habitats or conditions^1^,^2^. Prime evidence for adaptation is the maintenance and reproducibility of latitudinal clines in the frequency of chromosomal inversions observed in continuous populations of several species of drosophilids across continents^3^,^4^,^5^,^6^. In particular, *Drosophila subobscura* has been studied extensively due to its abundant inversion polymorphism and its recent invasion of large areas of North and South America. The frequencies of most chromosomal inversions in *D. subobscura* are correlated with latitude in the Palearctic region. In addition, similar latitudinal clines were also detected in both colonized American regions, suggesting that the geographic distribution of its inversion polymorphism is adaptive and not a mere consequence of historic events^3^,^7^,^8^. The chromosomal arrangements of *D. subobscura* are formed by single and overlapped inversions that may differentially affect levels of nucleotide variability, since the overlapped inversions may effectively reduce recombination. Previous studies focused on chromosomal inversions located in the segment I of the O chromosome (Fig. 1) and the A chromosome^9^,^10^,^11^,^12^,^13^, but segment II of the O chromosome remains largely unexplored despite bearing some chromosomal inversions whose frequencies cycle seasonally and respond to acute environmental events^14^.

The three chromosomal arrangements analysed in the present study (O_3+4_, O_3+4+1_ and O_3+4+7_) differ by the presence of single chromosomal inversions in the segment II of the O chromosome (Fig. 1). They can be considered medium size inversions since O_1_ includes 6.09 Mb and O_7_ inversion 11.76 Mb (estimated as in Pegueroles *et al*.^15^). The three arrangements are negatively correlated with latitude in the Palearctic region^16^, and one of them (O_3+4+7_) shows seasonal fluctuations^17^. These arrangements are found in sympatry in some regions around the Mediterranean Sea, although with different abundances^8^. The samples for the present research are from two well-studied localities, Barcelona and Mt. Parnes, where these arrangements coexist^18^.

Our aim is to test whether selection or drift are the evolutionary forces shaping genetic variability in single medium-size chromosomal inversions. We inferred population recombination and analysed patterns of DNA variation and linkage disequilibrium in six gene segments located within inverted and non-inverted regions, taking into account the age, the length of the inverted regions and the distance to the inversion breakpoint. We also evaluated whether variability patterns fit selectively neutral expectations using both evolutionary and protein structural approaches. We found significant genetic differentiation between arrangements despite extensive recombination being detected inside the inversions. Interestingly, we found nonsynonymous substitutions at the *Atpα* gene outside the inverted regions that appear to have been fixed by positive selection in association with both the O_3+4+1_ and O_3+4+7_ arrangements, and that occur at residues in the structure of the ATPase α subunit which are known to confer resistance to the plant toxin ouabain.

## Results

### Nucleotide variation and genetic differentiation

To characterize the genetic content of the O_3+4+1_ and the O_3+4+7_ arrangements, we first calculated nucleotide variation and divergence for each of the six gene fragments studied (Table 1). Sequences for O_3+4_ individuals are from Pegueroles *et al*.^11^ and diversity estimates are reported herein to facilitate comparison. The number of haplotypes was approximately the same as the number of analysed lines, except for *Sqd* and *Atpα* genes in O_3+4+1_ arrangement that had lower numbers of haplotypes (Table 1). Considering the variability of the O_3+4_ arrangement as a baseline, we observed a decrease in variability in the intronic regions of *Sqd* and *Atpα* genes at both inverted arrangements (Fig. 2). In contrast, we observed increased variability in the first exonic region of *Atpα* gene for the O_3+4+7_ arrangement. Diversity levels were highly variable between genes (Table 1, Fig. 2). The intronic regions of the *Pif1A* gene showed the highest diversity (π) for all three arrangements despite being located within the O_7_ inversion. The *Yrt* gene also showed high π levels despite most of the amplified fragment being exonic and located close to the O_1_ breakpoint and outside the inversion. Since the proportion of intronic-exonic regions amplified varied among genes, genetic variability was also estimated in silent positions exclusively (i.e., both synonymous and non-coding sites) to avoid biases in diversity estimates (Table 1). In agreement with π results, silent nucleotide variability (π_sil_) remained highly variable among genes, but quite similar when comparing the two inverted arrangements. No relationship was detected between π and distance to breakpoints, since Pearson correlation values were negative and non-significant for all arrangements.

Most genes located inside the inverted regions showed significant genetic differentiation between arrangements (Table 2). However, the largest significant *F*_ST_ values were found for the *Atpα* gene, which is located close to but outside the studied inversions. Since recombination is expected to be constrained near inversion breakpoints, we plotted genetic differentiation with respect to the distance to nearest breakpoint. No relationship between genetic differentiation and distance to the nearest inversion breakpoint was found (Pearson correlation values were negative and non-significant) with most of the genes having similar *F*_ST_ values regardless of their location within or outside the inverted region (Suppl. Fig. 1).

### Linkage disequilibrium, gene flux and age of the inversions

If chromosomal inversions are effectively reducing recombination levels, we would expect higher levels of LD within and between genes located inside them. However, levels of LD in these genes were very low and only a strong LD was observed within the *Atpα* gene (Fig. 3A). Significant associations after adjusting for multiple testing (in green) were obtained only within genes and never between genes regardless of their location in relation to the inversions (Fig. 3B–D). The highest ZnS values were obtained for the *Atpα* gene with 0.34 and 0.26 when analyzing together inversions O_3+4_–O_3+4+1_ and O_3+4_–O_3+4+7_, respectively (Table S1). Surprisingly, ZnS values were in general higher for O_3+4+1_ and O_3+4+7_ sequences alone, than compared to the O_3+4_ arrangement, suggesting the presence of recombination events between arrangements.

The low levels of linkage disequilibrium detected within inverted regions suggest that recombination between chromosomal arrangements may be frequent. Recombination was detected within and between arrangements for all genes (Rho, Table S1). Surprisingly, recombination estimates were higher when comparing different chromosomal arrangements than when comparing the same inversion. This result may simply be due to the higher number of informative sites when combining arrangements. Some gene conversion tracts (GCTs) were detected (Table S2) despite probabilities of a site to be informative for gene conversion events (*ψ* values) are low (10^−3^ to 10^−4^). A total of five and eight tracts were observed between O_3+4_–O_3+4+1_ and O_3+4_–O_3+4+7_, respectively. The lengths of the tracts were highly variable, ranging from 7 to 1573 bp, and the largest tracts were found in the *Atpα* gene. Since GCTs are expected to be small, the large tracts observed might be due to single or double crossover events, given that *Atpα* gene is located outside the inversion.

Sequence networks for all genes were highly reticulated, with the exception of the *Atpα* gene, suggesting high levels of recombination among individuals carrying different chromosomal arrangements for genes located inside and outside inversions (Suppl. Fig. 2). For the *Sqd* gene, despite being located within all three inversions and presenting significant *Fst* values between arrangements (Table 2), individual sequences of the same arrangement seldom clustered together suggesting high rates of exchange among chromosomal arrangements (Suppl. Fig. 2). For the *Atpα* gene, it was possible to distinguish three clades corresponding to each arrangement, although four recombinant individuals (FMP2, FBC49, FBC76 and MP36) could be identified matching those detected as GCT (Table S3). Two of them have the O_3+4_ arrangement (FMP2 and FBC49) and a GCT length larger than 1422 bp (Table S2), FBC76 has the O_3+4+7_ arrangement and also a large GCT (1573bp) and MP36 has the O_3+4+1_ arrangement and a small GCT (52 bp). In addition, for the *Atpα* gene, the number of recombination connections within O_3+4+1_ and O_3+4+7_ arrangements was lower than within the O_3+4_ arrangement (Suppl. Fig. 2), which could indicate a more recent origin of the former two arrangements.

**Figure 1:**
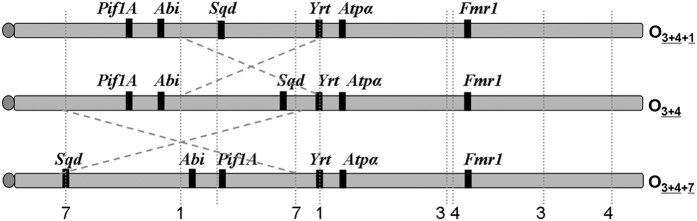


**Figure 2:**
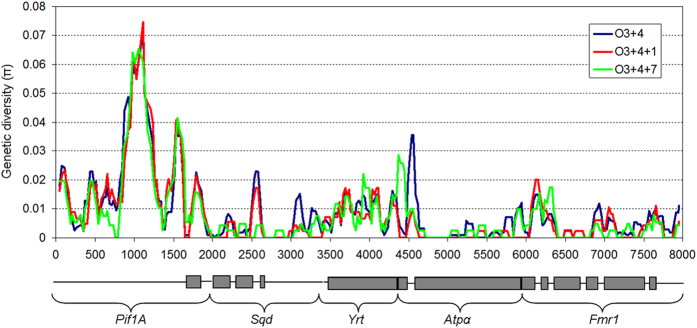
Genetic diversity (π) for the O_3+4_, O_3+4+1_ and O_3+4+7_ chromosomal arrangements using the concatenated genes data set. Grey boxes and solid lines underneath mark exonic and intronic regions, respectively.

**Figure 3:**
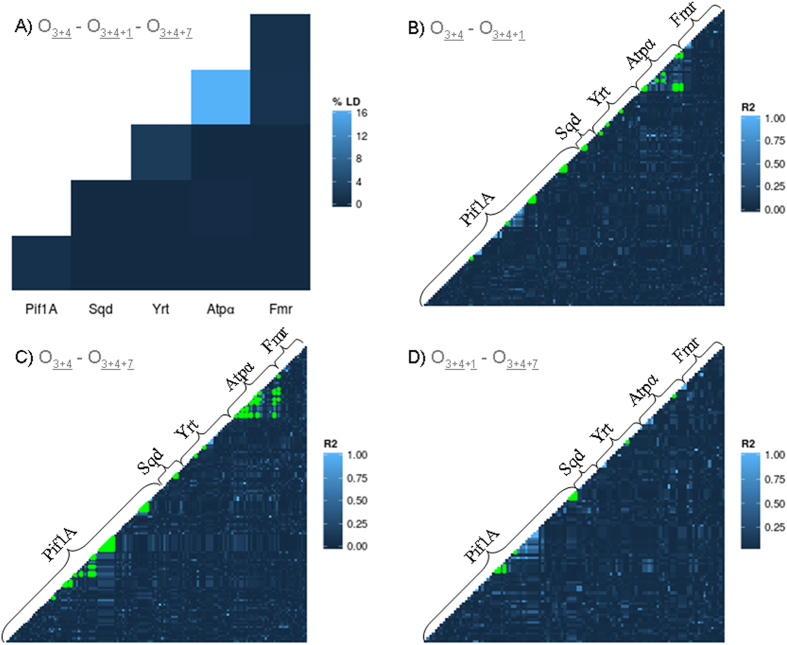
(**A**) Percentage of significant LD among O_3+4_, O_3+4+1_ and O_3+4+7_arrangements within and between genes. (**B–D**) Pairwise LD measured as R2 for O_3+4+7_–O_3+4_, O_3+4+1_–O_3+4_ and O_3+4+1_–O_3+4+7_ comparisons. Green dots correspond to the significant associations after adjusting for multiple comparisons using Benjamini and Hochberg method (1995). Gene order in O_3+4_ and O_3+4+1_ is the same (Fig. 1) and has been used for homogeneity in all comparisons.

Inversion ages may be overestimated from genes located in central positions of inverted regions, since they are more prone to be included in double crossovers consequently introducing additional variation from other arrangements. We estimated the age of the O_3+4+7_ arrangement using the *Sqd* gene, which is the closest to the proximal breakpoint of the O_7_ inversion (Fig. 1), to be 0.47 ± 0.12 Myr assuming that the divergence time between *D. subobscura* and *D. pseudoobscura* is 17.7 ± 4.4 Myr^19^. The age of the O_3+4+1_ arrangement was estimated to be 0.52 ± 0.13 Myr using the *Sqd* gene and the same divergence time. BEAST program could not be used to estimate the age of those two inversions due to the high recombination detected among the three arrangements (Suppl. Fig. 2).

### Test of neutrality and adaptive evolution

To evaluate whether any of the six genes are under positive selection, we performed several statistical tests for departure from the expectations of an equilibrium neutral model of evolution. A majority (93%) of the Tajima’s D and Fu and Li’s D test statistics were negative (Table S4). These overall results suggest a general trend towards an excess of low frequency polymorphisms that could be due to population growth. It is worth noting that Tajima’s D and Fu and Li’s D test were only significant for the the *Atpα* gene in the O_3+4+1_ arrangement when using all positions (Table S4), although both tests failed to detect significant departures from neutrality when excluding the recombinant individual MP36 and/or using only silent sites (Table S5). In contrast, Tajima’s D test was statistically significant for *Atpα* in the O_3+4+7_ arrangement when excluding recombinant individuals but not when using silent sites only (Table S5), raising the possibility of negative selection on polymorphic amino acid replacements at this gene.

The McDonald and Kreitman test, which contrasts nonsynonymous and synonymous polymorphism and divergence, was only significant for the *Atpα* gene in the O_3+4+7_ arrangement (*P *= 0.0003). For this gene, the number of polymorphic sites was 9 (7 nonsynonymous and 2 synonymous), while the number of differences between species was 60 (9 nonsynonymous and 51 synonymous, Table S6, Supporting information). The Direction of Selection (DoS) statistic is −0.442 for the O_3+4+7_ arrangement of this gene (Table S6, Supporting information). If one assumes synonymous sites are neutral, then this pattern would indicate an excess of nonsynonymous polymorphism present at the *Atpα* gene within the O_3+4+7_ arrangement. Certain amino acid changes nearly fixed in this arrangement (99, 109, 111 and 122 probably implicated in resistance to plant toxins, see below) could be maintained by diversifying selection as polymorphisms, while the rest are low frequency variants that could be weakly deleterious and kept at low frequencies by negative selection.

**Figure 4:**
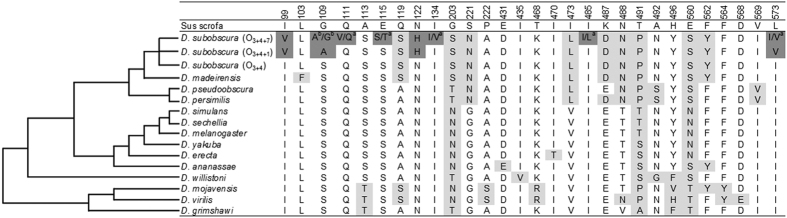
Amino acid replacements detected in the amplified region of the *Atpα* gene in 14 *Drosophila* species. ^a^low frequency amino acid (<0.3); ^b^equal frequency amino acid. Numbering corresponds to the mature pig enzyme (GenBank #: 1 × 03938). Shading is as follows: Pale grey: amino acid changes that occurred in the O_3+4_ arrangement of *D. subobscura* or in any other Drosophila species. Dark grey: changes found only in the O_3+4+1_ and/or O_3+4+7_ arrangements in comparison to the other Drosophila species.

**Figure 5:**
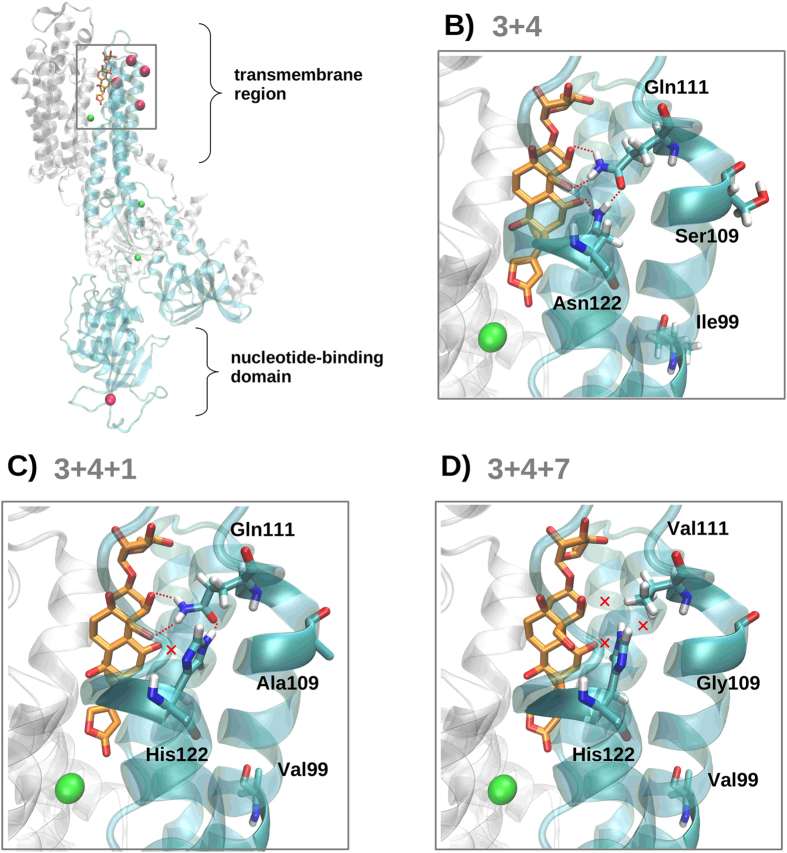
(**A**) Structural model of the ATPase α-subunit. The gene region sequenced is depicted in cyan and ouabain in orange sticks. Pink spheres represent positions presenting variation between arrangements and green spheres show the ion positions derived from the crystal structure of the template. (**B–D**) Detailed view of mutations located in the transmembrane region of O_3+4_, O_3+4+1_ and O_3+4+7_ arrangements. Hydrogen bonding interactions are represented as red dotted lines, while lack of these interactions is signalled with a red cross.

We tested for long-term positive selection at the *Atpα* gene using several site and branch-site tests implemented in CodeML of the PAML v4 package^20^, that were based on the consensus sequences of the arrangements (see methods). All positions in the consensus sequences correspond to nearly fixed substitutions between lineages except for amino acid position 109 in the O_3+4+7_ arrangement (Fig. 4) where the two equally likely substitutions (A/G) were evaluated separately. Site tests of the entire gene fragment (M1a vs M2a and M7 vs M8, see Materials and Methods section), which assume that the strength and direction of selection is uniform across all lineages, failed to detect positively selected sites in the *Atpα* gene (Table S7). However, the branch-site test 2, that allows to detect sites that evolved under positive selection in an specific lineage, inferred positive selection for several codons on the O_3+4+7_ arrangement regardless of which amino acid is present in position 109 (Table S7), but not on the O_3+4_ and O_3+4+1_ arrangements. Positions that showed departures from neutrality according to PAML in the O_3+4+7_ arrangement are 99, 109, 111 and 122, which are the positions highlighted in the structural protein model (Fig. 5). In fact, positions 111 and 122 are the ones affecting the ouabain–*Atpα* interaction (see below), and the nonsynonymous change at position 111 was only found in O_3+4+7_. Using BEAST, after removing recombinants, the time to most recent common ancestor (TMRCA) was estimated in 0.21 ± 0.009 Myr (mean ± SE) for O_3+4+1_, 0.46 ± 0.018 Myr for O_3+4+7_ and 1.69 ± 0.05 Myr for all O_3+4_. Using the average silent nucleotide diversity the time to most recent common ancestor (TMRCA) was estimated in 0.09 ± 0.02 Myr and 0.13 ± 0.03 Myr for O_3+4+1_ and O_3+4+7_ respectively considering that the divergence time between *D. subobscura* and *D. pseudoobscura* is 17.7 ± 4.4 Myr^19^.

We checked whether the variability in the *Atpα* gene, and specifically the nonsynonymous changes, were already present in other drosophilids or appeared *de novo* in *D. subobscura*. Amino acid replacements observed within the amplified region of the *Atpα* gene among 14 *Drosophila* species are summarized in Fig. 4. Most of the replacements detected can be assigned to specific lineages. For instance, a G to N replacement at position 221 and V to L replacement at position 473 were both detected for all species of the *obscura* group. Interestingly, some changes were only detected in O_3+4+1_ and O_3+4+7_ arrangements and not in any other of the thirteen drosophilids studied neither in the O_3+4_ arrangement of *D. subobscura*. These changes can be classified in three groups: shared mutations between the two arrangements (positions 99, 109 and 122), specific high frequency mutations in one arrangement (position 111 for O_3+4+7_ and 573 for O_3+4+1_), and specific low frequency mutations (115, 134, 485, 573 for the O_3+4+7_). The two equally frequent polymorphic amino acids in position 109 of the O_3+4+7_ arrangement (A and G) are small and non-polar, while the ancestral amino acid was polar (S) (Fig. 4). The O_3+4_ arrangement of *D. subobscura* is more similar to (closely) related species (i.e. *D. madeirensis*) than to O_3+4+1_ and O_3+4+7_ arrangements.

### Three-dimensional structure of the ATPase α-subunit and putative functional consequences

At least two of the amino acid replacements observed in the ATPase α-subunit may impact the binding of the cardenolide ouabain, a plant toxin capable of blocking ATPases^21^,^22^. According to the crystallized ouabain–Na^+^, K^+^-ATPase complex^23^, ouabain would interact with a set of hydrophobic residues in helices αM4 and αM5 and would establish particular polar interactions with helices αM1, αM2 and αM6. Helices αM1, αM2 were sequenced in the present work and are depicted in cyan in Fig. 5. The positions contributing to variation between arrangements are located both in the transmembrane region of the protein as well as in its nucleotide-binding domain (depicted as magenta spheres in Fig. 5A). Of the variants located in the transmembrane region, we can expect different levels of impact regarding ouabain binding. Changes in positions 99 and 573 (I99 V and I573 V), which are located in the transmembrane and nucleotide-binding domain respectively, are similar in terms of hydrophobicity and shape and are not expected to have a big impact in terms of protein function. In the case of amino acid position 109 of the transmembrane segment, amino acid replacement results in a loss of a polar residue that could indirectly affect protein stability and insertion in the lipid bilayer (S109A in O_3+4+1_ and S109A, G in the O_3+4+7_ arrangement, Fig. 5B–D). Interestingly, we detected two further changes in the transmembrane region that could directly affect the binding of ouabain to the Atpα protein. In the O_3+4_ arrangement, ouabain establishes stabilizing hydrogen bonding interactions with residues Gln111 and Asn122 of the alpha subunit of the ATPase (Fig. 5B, red dashed lines). These interactions are further stabilized by a hydrogen bond between these two residues. In contrast, the replacement of Asn122 by His122 in both O_3+4+1_ and O_3+4+7_ arrangements destroys the interaction between this residue and ouabain (Fig. 5C, see red cross). In addition, mutation from Gln111 to Val111 in the O_3+4+7_ arrangement destroys the second stabilizing interaction as well as the intramolecular hydrogen bond formed by *Atpα* residues (Fig. 5D, see red crosses).

## Discussion

Chromosomal inversions are known to strongly influence patterns of genetic diversity within their breakpoints. The degree of inversion variability and differentiation depends on the time since the formation of the inversion, on its size (large inversions are more likely to have double-crossovers within them), and on selection pressure^4^,^24^,^25^,^26^. Genetic differentiation for O_3+4+1_ and O_3+4+7_ arrangements was significant despite variability levels for most of the genes located within them seem to have recovered to the level observed in the O_3+4_ ancestral arrangement. We estimated the age of these derived inversions considering that the *Sqd* gene is roughly 0.50 Myr (assuming that the divergence time between *D. subobscura* and *D. pseudoobscura* is 17.7 ± 4.4 Myr)^19^. The age of the O_3+4_ arrangement was estimated to be 0.90 Myr using the same divergence time as mentioned above^11^. Thus, as expected, the O_3+4+1_ and O_3+4+7_ arrangements are younger than O_3+4_ from which they most likely derived (Fig. 1). However, the *Sqd* gene network showed recombination connections between different arrangements, further preventing estimating inversion ages using the coalescent process, which may result in an overestimation of their age. It is worth noting that the estimation of inversion ages may vary between markers^9^,^11^,^27^,^28^. Thus, our results should be interpreted with caution until more markers are available to confirm them.

The genetic differentiation that we found between O_3+4+1_–O_3+4_ and O_3+4+7_–O_3+4_ arrangements was smaller than between the older O_3+4_–O_ST_ arrangements^9^,^11^,^27^. The presence of two overlapped inversions (O_3_ and O_4_) in the later comparison may prevent crossovers formation more efficiently due to physical constraints. Overlapped inversions may be an important non-selective factor modulating nucleotide variability patterns and their absence may facilitate recombination. The small *F*_ST_ values obtained for individual genes located within O_1_ and O_7_ inverted regions suggest the presence of frequent genetic exchange with non-inverted arrangements for these regions, supporting recombination as the main contributor to variability recovery^24^.

We did not detect a significant relationship between genetic variability and distance to breakpoints, as observed in previous studies of *D. subobscura*^9^,^10^, *D. buzzatti*^29^, and *Anopheles gambiae*^30^. However, for *D. melanogaster* mixed results are obtained depending on the inversions and populations of origin evaluated, with peaks of high and low variability and differentiation interspersed^31^,^32^. We find that genetic differentiation close to inversion breakpoints can also be eroded through time at a gene specific rate, supporting previous experimental studies in *D. subobscura*^15^, and contrasting with those obtained in *D. pseudoobscura*^33^. As expected by the presence of high levels of recombination, linkage disequilibrium levels were low within inversions. Our results contrast with those obtained in *D. pseudoobscura* inversions, which generally show high levels of LD between genes associated with inversions that have been interpreted as an evidence for epistasis^6^,^34^. For *D. melanogaster* strong LD within the region spanned by In(3R)Payne has been detected although it is not uniformly distributed^31^. According to tests of neutrality based on frequency distributions (Tajima’s D and Fay and Wu’s H), there was a tendency towards an excess of low frequency polymorphisms for all genes consistent with a recent expansion of the species^35^, but only the *Atpα* gene departed from neutral expectations. Taken altogether, variability in all genes (with the only exception of the *Atpα* gene) in the O_3+4+1_ and O_3+4+7_ arrangements seem to be shaped mainly by extensive recombination rather than Darwinian (positive) selection.

Our results indicate that variability patterns of the *Atpα* gene seem to be strongly influenced by natural selection in the O_3+4+1_ and the O_3+4+7_ arrangements. Despite being located outside both studied inversions (but less than 2 Mb apart from the closest inversion breakpoint), we detected high levels of genetic differentiation when compared to the ancestral O_3+4_ arrangement due to fixed nonsynonymous differences. In *D. melanogaster* parallel geographic variation in regions inside and outside inversions have been observed across continents^36^. Besides that, SNPs varying in frequency seasonally throughout *D. melanogaster* genome–and not exclusively concentrated in inversions–have also been described^37^. Thus, spatial and temporal varying selection seems also to strongly influence regions outside inversions. Differences in the *Atpα* gene between O_3+4+1_ and O_3+4+7_ arrangements were significant at the nucleotide level although at the amino acid level, both arrangements are nearly identical. Interestingly, all specific changes of the *D. subobscura* lineage occurred in the O_3+4+1_ and/or O_3+4+7_ arrangements, since changes detected in the O_3+4_ arrangement were shared with *D. madeirensis,* which is consistent with their common ancestry. In addition, protein sequences of O_3+4_ and O_ST_ arrangements of *D. subobscura* were reported to be identical^11^.

The nature of natural selection acting on the *Atpα* gene is quite complex. The McDonald-Kreitman test was significant for *Atpα* gene and the Direction of Selection (DoS) statistic was negative in a direction consistent with strong selective constraint acting on most of the protein. These results are in agreement with the essential nature of this gene for individual survival. Nonetheless, PAML did reveal significant evidence for positive selection acting on several codons within the *Atpα* gene, including amino acid replacements at two codons that would confer resistance to a plant toxin. Positive selection acting on highly conserved genes has also been reported in other studies: according to Pupko & Galtier^38^, primate mitochondrial genomes evolved through episodes of positive selection at a few sites, enabling the fine-tuning of the three-dimensional protein structure to optimize the function of conserved genes. Similarly, Vasseur *et al*.^39^ found rare alleles with evidence of positive selection in some genes of the NLR family although this family is under strong purifying selection due to its vital role.

The case of the *Atpα* gene indicates that positive selection is able to act within a highly conserved gene to maintain adaptive mutations associated with certain chromosomal inversions. The structural analysis of the ouabain-ATPase α-subunit complex shows that two substitutions, both in the O_3+4+1_ and the O_3+4+7_ arrangements (111V and 122H), would reduce the affinity of the ATPase complex to bind the cardenolide ouabain due to the destruction of stabilizing hydrogen bonds. Remarkably, these observations are in line with mutagenesis studies showing a significantly increased survival of cells transfected with constructs having mutations 111V and 122H (from *D. melanogaster*) after ouabain treatment, and a 2.250-fold increased resistance to this toxin when bearing both mutations^21^,^40^. Previous studies demonstrated that adaptive mutations in Na,K-ATPase, such the ones in positions 111 and 122, were acquired in parallel in some cardenolide-feeding species^21^,^22^. Three hypotheses could explain the presence of convergent mutations in O_3+4+1_ and O_3+4+7_ arrangements in *D. subobscura*. (1) In a parallel scenario^41^, mutations may have occurred independently in the two new arrangements as the result of adaptation to similar environmental conditions. (2) In a collateral scenario^41^, variants from an ancestral polymorphism could have been independently captured during the formation of the two inversions and subsequently been maintained by selection. (3) Finally, amino acid substitutions that occurred in one of the two arrangements in response to selection could have been subsequently acquired by the other inversion through double recombination or gene conversion between arrangements, with those variants being subsequently driven to high frequency in both arrangements due to similar selective pressures. All three of these possible scenarios include natural selection and suggest that epistatic interactions between the *ATPα* gene and genes located inside both inversions (O_1_ and O_7_) are necessary to account for the maintenance of amino acid similarities despite *ATPα* gene being located outside both inversions. Furthermore the reduced number of recombinants with O_3+4_ can only be explained by selection if recombinant individuals are effectively purged from populations to maintain adaptive interactions. Currently available data does not allow us to discriminate between these three scenarios although the collateral hypothesis seems less likely since in *D. madeirensis*, O_3+4_ and O_ST_ share almost identical amino acid composition. Given the high chromosomal polymorphism in *D. subobscura* and the many inversion breakpoints in the neighbouring area of the *Atpα* gene^8^, future analysis of other chromosomal arrangements may help to reconstruct the process of acquisition of these adaptive substitutions, and to determine whether they were already present in a common ancestor (i.e. synapomorphy) or acquired by parallel evolution or through recombination.

Cardenolides have a huge diversity of chemical forms and are sporadically distributed across 12 families of angiosperms^42^. Cardenolide feeding species have been typically associated with plants of the family Apocynaceae, notably in the genera *Asclepias* and *Apocynum*^21^,^22^. *Asclepias* has a Neartic distribution and *Apocynum* a temperate Northern hemisphere distribution^43^, and cardenolides production seems to form latitudinal clines of different sign depending on the *Asclepias* species^42^,^44^. *D. subobscura* is a generalist saprophytic insect and its diet includes decaying plant material and fruits, fungi, yeast and microbials^45^, and it is known to be able to feed from decaying *Digitalis purpurea*^46^, a plant containing ouabain. We hypothesize that the appearance of mutations in the O_3+4+7_ and O_3+4+1_ arrangements conferring the ability to feed on cardenolide containing plants has changed the fitness of associated chromosomal inversions resulting in nonsynonymous polymorphism. Thus, in certain environments (i. e. in the presence of toxic plants) positive selection will favour the maintenance of adaptive variants. Future studies may help elucidate whether the observation of adaptive mutations in some arrangements of *D. subobscura* reflects geographical distribution of cardenolide-containing plants in the Mediterranean region and confirm whether these amino acid substitutions confer resistance to cardenolides in these insects.

## Materials and Methods

### Fly samples and DNA sequencing

A total of 45 isochromosomal lines for the O chromosome of *D. subobscura* derived in Araúz *et al*.^18^ were used: 11 O_3+4+1_ and 12 O_3+4_ lines from Mt. Parnes (Greece) and 10 O_3+4+7_ and 12 O_3+4_ lines from Barcelona (Spain). Genes were selected according to their chromosomal location within or nearby the studied inversions (Fig. 1). The six genes are *Pif1A* (PFTAIRE-interacting factor 1A), *Abi* (Abelson interacting protein), *Sqd* (Squid), *Yrt* (Yurt), *Atpα* (Na pump α subunit) and *Fmr1* (Fragile X mental retardation). Genomic DNA extraction, DNA amplification and sequencing reactions for the O_3+4+1_ and O_3+4+7_ arrangements were carried out as reported in Pegueroles *et al*.^11^. Sequencing was done on a 3730 Analyzer (Applied Biosystems) at the Serveis Cientifico-Tècnics from Universitat de Barcelona. Sequences were assembled with SeqMan II (DNASTAR) and multiply aligned with Clustal W^47^ implemented in BioEdit v7^48^. *S*equences for the O_3+4+7_ and O_3+4+1_ arrangements are available at GenBank under the accession numbers KT318937- KT319043. Sequences for the O_3+4_ arrangement of *D. subobscura* and *D. madeirensis* were obtained from GenBank (accession # JN882382-JN882400, JN882406-JN882429, JN882441-JN882461, JN882472-JN882495, JN882508-JN882529, JN882541- JN882564 and JN882376-JN882381). Sequences from the other 12 *Drosophila* species with sequenced genome were downloaded from Flybase database (http://flybase.org).

### Nucleotide polymorphism and genetic differentiation

Nucleotide polymorphism and genetic differentiation were estimated with DnaSP v5^49^. We calculated the standard parameters of molecular diversity: number of haplotypes (*h*), number of polymorphic sites (*S*) and number of singletons, nucleotide diversity (π)^50^, nucleotide diversity in synonymous sites and non-coding positions (π_sil_)^51^, silent site heterozygosity (θ_sil_)^52^ and divergence per silent site between *D. subobscura* and *D. pseudoobscura* (*K*_sil_)^51^. Due to the presence of duplications in the *Abi* gene (data not shown), very few individuals could be sequenced for the O_3+4+1_ arrangement. Thus, the concatenated data set does not contain *Abi* gene sequences. Overall, 21 O_3+4_, 10 O_3+4+1_ and 8 O_3+4+7_ chromosomes were included in the concatenated data set and genes combined with Concatenator v1^53^. Nucleotide diversity (π) across the concatenated data was calculated using a sliding window of 100 nucleotides with a step size of 25. Genetic distances were computed with *F*_ST_^54^ and *S*nn^55^ and its significance estimated with 10,000 replicates. The distance of each gene to the nearest inversion breakpoint in bp was calculated assuming that all cytological bands contain the same genetic content and the length of the O chromosome of *D. subobscura*, but not its gene order^56^, is equivalent to that of the chromosome 2 of *D. pseudoobscura* as in Pegueroles *et al*.^57^.

### Neutrality tests

Tajima’s D^58^ and Fu and Li’s D^59^ tests were carried out to assess whether the site frequency spectrum of variation within arrangements differ from their expectation under an equilibrium neutral model, using *D. pseudoobscura* as an outgroup. This species was used as outgroup instead of *D. madeirensis* since the level of divergence to *D. subobscura* for the latter is too low for these genes^11^. Furthermore, to test for footprints of selection we performed the McDonald and Kreitman^60^ test, the Direction of Selection (DoS) statistic^61^, and several site and branch-site tests implemented in CodeML of the PAML v4 package^20^. Site tests of the entire gene, allowing the ω ratio to vary among sites, were performed comparing two pairs of models, the nearly neutral model M1a (model* *= 0; NSsites* *= 1) with the alternative positive selection model M2a (model* *= 0; NSsites* *= 2), and the neutral model M7 (model* *= 0; NSsites* *= 7, ncatG* *= 10) with the alternative selection model M8 (model* *= 0; NSsites* *= 8, ncatG* *= 10). For the branch-site test 2, aiming to detect positive selection affecting a few sites, in the neutral model we used the parameters model* *= 2, NSsites* *= 2, fix_omega * *=  1 and omega* *= 1. and for the alternative selection model we used model* *= 2, NSsites* *= 2, fix_omega = 0 and omega* *= 1.5. All these tests were applied to the *Atpα* gene after excluding recombinant individuals and using the consensus sequences of the O_3+4_, O_3+4+1_ and O_3+4+7_ arrangements of *D. subobscura* with both *D. madeirensis* and *D. pseudoobscura* sequences as outgroups. Neutral and alternative models were compared using a likelihood ratio test and the P-value was assessed using a chi-squared test.

### Linkage disequilibrium and recombination

For the concatenated data set we estimated the percentage of pairwise comparisons between informative sites presenting significant linkage disequilibrium (LD), and their statistical significance was analysed with Fisher’s exact test implemented in DnaSP v5^50^. P-values were adjusted for multiple testing using the false discovery rate method of Benjamini & Hochberg^62^. LD between pairs of polymorphic sites was also measured with r^2^ parameter^63^ and *ZnS*^64^ as a global measure of LD obtained with DnaSP. LD plots were performed using ggplot2 package^65^. The population recombination rate (ρ = 4N_e_r, where N_e_ is the effective population size and r is the rate of recombination) was estimated using a composite likelihood method^66^ computed with LDhat v2.1 (http://www.stats.ox.ac.uk/~mcvean/LDhat/instructions.html). Recombination networks were constructed using SplitsTree4 program^67^. Gene conversion tracts (GCT) were identified using the method of Betrán *et al*.^68^ implemented in DnaSP. In order to avoid confounding effects due to the population of origin, *F*_ST_, ρ, GCT and LD parameters were calculated between arrangements from the same population, despite the lack of genetic differentiation observed between O_3+4_ arrangements from different populations^11^.

### Age of inversions

The ages of inversions were estimated for the *Sqd* gene, since it is located inside the inverted regions and close to the breakpoint (Fig. 1), using the average silent nucleotide diversity within inversions and excluding individuals carrying gene conversion tracts^10^,^11^,^27^. The number of substitutions per site per year was calculated using the divergence per silent site between *D. subobscura* and *D. pseudoobscura*, based on our sequences and using divergence time of 17.7 ± 4.4 Myr^19^. We dated the time to the most recent ancestor (TMRCA) for the O_3+4+1_ and O_3+4+7_ arrangements of the *Atpα* gene using the same method and also using BEAST 1.8.0^69^. We used a lognormal relaxed clock model and considered the same divergence time and a mutation rate of 0.011 estimated for Drosophila species based on 176 nuclear genes^19^. The substitution model used was HKY + G + I, being the best substitution model for the *Atpα* gene inferred with jModelTest 2.0^70^,^71^, with runs of 2 million steps, sampling a tree every 200 steps. Tracer v1.6^72^ was used to check convergence of parameters and to obtain mean and standard errors (SE) of the time to the most common ancestor of all sequences for a given inversion. We discarded 10% of the steps as burn-in. In both methods we did not include recombinant individuals MP36 and FBC76.

### Structural analysis of the Na^+^,K^+^-ATPase–ouabain complex

The crystal structure of a high-affinity Na^+^,K^+^-ATPase–ouabain complex (PDB ID 4HYT), which shows a 74% amino acid sequence identity with the predicted ATPα protein for *D. subobscura*, was selected for homology modelling. The model was built using the MOE package (http://www.chemcomp.com/software.htm). After sequence alignment (default settings), ten models were generated using the Amber12:EHT force field^73^. The best model for each arrangement was selected and superposed on the PDB ID 4HYT crystal structure in order to align the cardenolide ouabain, a plant toxin capable of blocking ATPases, to the newly obtained homology models. The resulting ouabain-receptor complexes were further refined by performing an energy minimization of ouabain and its binding pocket (defined as all residues at 4.5 Å of the compound) using the Amber12:EHT force field by applying gradient minimization until the RMS gradient was lower than 0.001 kcal mol^−1^ Å^−1^. Representations of ouabain-receptor complexes were created using VMD 1.9.1^74^.

## Additional Information

**Accession codes:** D. subobscura sequences data are available at GenBank under the accessions numbers KT318937- KT319043.

**How to cite this article**: Pegueroles, C. *et al*. Inversions and adaptation to the plant toxin ouabain shape DNA sequence variation within and between chromosomal inversions of *Drosophila subobscura. Sci. Rep.* **6**, 23754; doi: 10.1038/srep23754 (2016).

## Supplementary Material

Supplementary Information

## Figures and Tables

**Table 1:** Nucleotide variation and divergence per chromosomal arrangement for the six genes studied.

Gene	Pop	Arrangement	*n*	*h*	*S*	Singletons	*π*	*π*_sil_	*θ*_sil_	*K*_sil_
***Pif1A***	**MP**	**O_3+4+1_**	9	9	104	61	0.021	0.022	0.025	0.188
		**O_3+4_**	12	12	120	55	0.021	0.023	0.026	0.191
	**BC**	**O_3+4+7_**	9	9	89	54	0.017	0.018	0.021	0.187
		**O_3+4_**	12	12	114	42	0.022	0.023	0.024	0.190
***Abi***	**MP**	**O_3+4+1_**	3	3	8	8	0.003	0.008	0.008	0.174
		**O_3+4_**	12	12	32	20	0.005	0.012	0.017	0.173
	**BC**	**O_3+4+7_**	10	9	33	21	0.006	0.015	0.018	0.172
		**O_3+4_**	7	7	24	15	0.006	0.014	0.015	0.173
***Sqd***	**MP**	**O_3+4+1_**	10	5	12	8	0.002	0.005	0.008	0.115
		**O_3+4_**	12	12	25	16	0.005	0.006	0.008	0.116
	**BC**	**O_3+4+7_**	10	10	17	12	0.003	0.004	0.006	0.115
		**O_3+4_**	10	10	21	14	0.005	0.006	0.007	0.115
***Yrt***	**MP**	**O_3+4+1_**	11	11	28	14	0.009	0.031	0.035	0.327
		**O_3+4_**	12	12	33	18	0.010	0.034	0.042	0.327
	**BC**	**O_3+4+7_**	9	9	31	18	0.010	0.035	0.013	0.325
		**O_3+4_**	12	12	35	21	0.010	0.035	0.041	0.326
***Atpα***	**MP**	**O_3+4+1_**	9	4	6	6	0.001	0.003	0.005	0.204
		**O_3+4_**	12	12	22	9	0.005	0.015	0.013	0.208
	**BC**	**O_3+4+7_**	9	8	22	17	0.004	0.006	0.010	0.204
		**O_3+4_**	12	11	27	16	0.005	0.016	0.019	0.209
***Fmr1***	**MP**	**O_3+4+1_**	10	10	30	24	0.004	0.007	0.010	0.150
		**O_3+4_**	11	11	38	25	0.005	0.009	0.012	0.151
	**BC**	**O_3+4+7_**	8	7	19	12	0.003	0.006	0.007	0.150
		**O_3+4_**	10	10	28	18	0.004	0.008	0.010	0.151

*Pop*, population: Mt. Parnes (MP) and Barcelona (BC); *n*, sample size; *h*, number of haplotypes; *S*, number of polymorphic sites; π, nucleotide diversity in all sites; π_sil_, nucleotide diversity in synonymous sites and non-coding positions; θ_sil_, heterozygosity in silent sites; *K*_sil_, divergence per silent site between *D. subobscura* and *D. pseudoobscura*.

**Table 2:** *F*_ST_ values for each gene and the concatenated set and the statistical significance of *S*nn (ns, not significant; 0.01 <*P < 0.05; 0.001 <**P < 0.01; ***P < 0.001).

	**O_3+4_ − O_3+4+1_**	**O_3+4_ − O_3+4+7_**	**O_3+4+1_ − O_3+4+7_**
*Pif1A*	−0.028 ns	0.146**	0.076 ns
*Abi*	–	0.065*	–
*Sqd*	0.082*	0.040*	0.071**
*Yrt*	0.017 ns	−0.001 ns	0.024 ns
*Atpα*	0.614***	0.525**	0.367***
*Fmr1*	−0.033 ns	0.004 ns	−0.005 ns
Concatenated	0.099*	0.182**	0.092*

Genes in grey are located inside inversions.
