# Supplementary material for: Inversions and adaptation to the plant toxin ouabain shape DNA sequence variation within and between chromosomal inversions of Drosophila subobscura
Source: Sci Rep. 2016 Mar 31;6:23754. doi: 10.1038/srep23754 (PMC4815013; doi:10.1038/srep23754)
Supplement: Supplementary Information [file srep23754-s1.doc]

**Inversions and adaptation to the plant toxin ouabain shape DNA sequence variation within and between chromosomal inversions of *Drosophila subobscura*.**

Cinta Pegueroles, Albert Ferrés-Coy, Maria Martí-Solano, Charles F Aquadro, Marta Pascual and Francesc Mestres

**
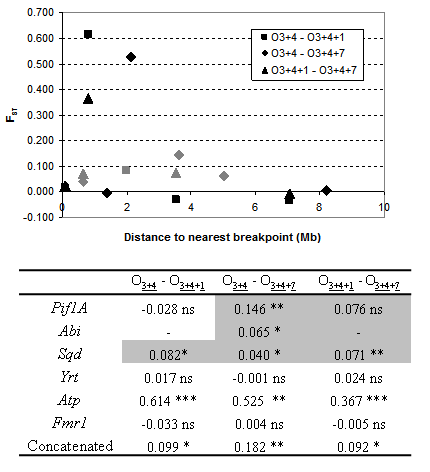
**

**Supplementary Figure 1:** Genetic differentiation (*F*_ST_) between chromosomal arrangements for each gene *vs* their distance to the nearest inversion breakpoint. Squares designate O_3+4+1_ - O_3+4_ comparisons, diamonds O_3+4+7_ - O_3+4_ and triangles O_3+4+1_ - O_3+4+7_. Grey symbols correspond to genes located inside inversions.


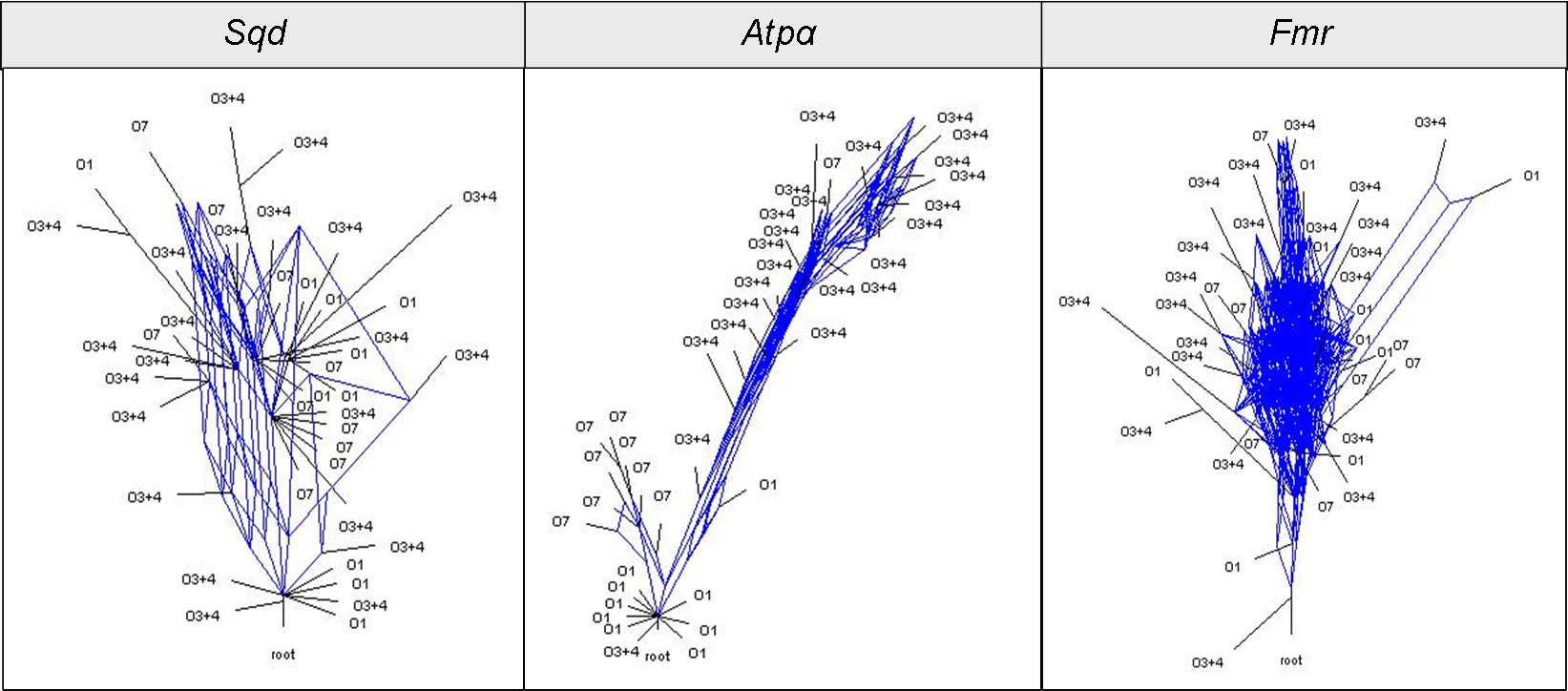
**Supplementary Figure 2:** Recombination networks constructed using SplitsTree4 program ^67^ for *Sqd* (a gene located within the inversions) and *Atpα* and *Fmr1* genes (both located outside the studied inversions) for individuals with O_3+4_ (O3+4), O_3+4+1_ (O1) and O_3+4+7_ (O7) arrangements.

**Table S1:** *Z*n*S* statistic values and the recombination parameter (Rho) *per* bp. Shadowed genes are located inside inversions. Zn*S* is not available for the *Atpα* gene in the O_3+4+1_ arrangement due to the lack of parsimony informative sites.

|  | **Arrangement** | **ZnS** | **Rho/length** |
| --- | --- | --- | --- |
| *Pif1A* | O_3+4+1_ | 0.172 | 0.055* |
|  | O_3+4+1_ - O_3+4_ | 0.066 | 0.055* |
|  | O_3+4+7_ | 0.178 | 0.030 |
|  | O_3+4+7_ - O_3+4_ | 0.094 | 0.055* |
| *Abi* | O_3+4+7_ | 0.327 | 0.008 |
|  | O_3+4+7_ - O_3+4_ | 0.144 | 0.013 |
| *Sqd* | O_3+4+1_ | 0.714 | 0.000 |
|  | O_3+4+1_ - O_3+4_ | 0.149 | 0.030 |
|  | O_3+4+7_ | 0.206 | 0.021 |
|  | O_3+4+7_ - O_3+4_ | 0.165 | 0.070* |
| *Yrt* | O_3+4+1_ | 0.195 | 0.046 |
|  | O_3+4+1_ - O_3+4_ | 0.090 | 0.110* |
|  | O_3+4+7_ | 0.182 | 0.067 |
|  | O_3+4+7_ - O_3+4_ | 0.078 | 0.110* |
| *Atpα* | O_3+4+1_ | n.a. | 0.009 |
|  | O_3+4+1_ - O_3+4_ | 0.335 | 0.007 |
|  | O_3+4+7_ | 0.200 | 0.000 |
|  | O_3+4+7_ - O_3+4_ | 0.262 | 0.003 |
| *Fmr1* | O_3+4+1_ | 0.089 | 0.004 |
|  | O_3+4+1_ - O_3+4_ | 0.089 | 0.033 |
|  | O_3+4+7_ | 0.365 | 0.006 |
|  | O_3+4+7_ - O_3+4_ | 0.114 | 0.012 |

* Rho per gene > 100

**Table S2:** Gene conversion tracts detected for each of the studied gene regions. The Ψ parameter is defined as the probability of a site to be informative for a gene conversion event. Shadowed genes are located inside inversions.

| **O_3+4_ - O_3+4+1_** | **Converted ^a^** | **Informative sites** | **ψ ^b^** | **Length (bp)** |
| --- | --- | --- | --- | --- |
| *Pif1A* |  | 1 | 0.00017 |  |
| *Sqd* |  | 3 | 0.00041 |  |
| *Yrt* | O_3+4_ | 4 | 0.00063 | 111 |
| *Atpα* | O_3+4_ | 17 | 0.00541 | 1573 |
| *Atpα* | O_3+4+1_ | 17 | 0.00541 | 52 |
| *Fmr1* |  | 0 | 0 |  |
| **O_3+4_ - O_3+4+7_** |  |  |  |  |
| *Pif1A* | O_3+4_ | 32 | 0.00464 | 466 |
| *Pif1A* | O_3+4+7_ | 32 | 0.00464 | 103 |
| *Pif1A* | O_3+4+7_ | 32 | 0.00464 | 208 |
| *Abi* |  | 1 | 0.00023 |  |
| *Sqd* | O_3+4+7_ | 3 | 0.00043 | 8 |
| *Yrt* | O_3+4+7_ | 2 | 0.00037 | 7 |
| *Atpα* | O_3+4_ | 18 | 0.00614 | 1422 |
| *Atpα* | O_3+4+7_ | 18 | 0.00614 | 1573 |
| *Fmr1* |  | 0 | 0 |  |

^a^ converted arrangement when identification was possible.

^b^ probability of a site being informative of a gene conversion event.

**Table S3:** Polymorphic sites among the 42 *D. subobscura* isochromosomal lines for the *Atpα* gene. Sequences are grouped according to their gene arrangement. Exonic regions appear shaded and boxes identify gene conversion tracts. The position number of each polymorphic site is in relation to the nucleotide alignment of *D. subobscura* sequences.

* nonsynonymous changes

**Table S4:** Tajima’s D and Fu and Li’s D neutrality tests for O_3+4+1_ and O_3+4+7_ chromosomal arrangements. Shadowed genes are located inside inversions.

|  |  | **Tajima's D** | **P-value** | **Fu and Li's D ^a^** | **P-value** |
| --- | --- | --- | --- | --- | --- |
| *Pif1A* | O_3+4+1_ | -0.654 | 0.262 | -0.972 | 0.223 |
|  | O_3+4+7_ | -0.581 | 0.277 | -0.990 | 0.211 |
| *Abi* | O_3+4+7_ | -0.899 | 0.194 | -0.794 | 0.248 |
| *Sqd* | O_3+4+1_ | -0.731 | 0.257 | -1.598 | 0.083 |
|  | O_3+4+7_ | -1.331 | 0.101 | -1.561 | 0.085 |
| *Yrt* | O_3+4+1_ | -0.684 | 0.253 | 0.118 | 0.550 |
|  | O_3+4+7_ | -0.888 | 0.219 | -0.789 | 0.275 |
| *Atpα* | O_3+4+1_ | -1.728 | 0.011 | -2.236 | 0.008 |
|  | O_3+4+7_ | -1.347 | 0.104 | -0.309 | 0.421 |
| *Fmr1* | O_3+4+1_ | -1.620 | 0.050 | -1.956 | 0.050 |
|  | O_3+4+7_ | -0.637 | 0.276 | -1.432 | 0.068 |

^a^ Fu and Li's D using *D. pseudoobscura* as an outgroup.

* P-values assessed by 1000 coalescent simulations

**Table S5:** Tajima’s D and Fu and Li’s D neutrality tests for the *Atpα* gene in the O_3+4+1_ and O_3+4+7_ chromosomal arrangements. Tests were performed including all positions, only silent sites, and including and excluding recombinant individuals.

| **Positions of the *Atpα* gene** | **Chromosomal arrangement** | **Tajima’s D** | **Fu and Li’s D** |
| --- | --- | --- | --- |
| all positions | O_3+4+1_ | -1.728* | -2.236* |
| all positions | O_3+4+7_ | -1.347 | -0.309 |
| silent sites | O_3+4+1_ | -1 | -2,236* |
| silent sites | O_3+4+7_ | -1.668 | -0.081 |
| all positions,  without recombinants | O_3+4+1_ | -1,310 | -1,677 |
| all positions,  without recombinants | O_3+4+7_ | -2.126* | -0.252 |
| silent sites,  without recombinants | O_3+4+1_ | -1.310 | -1.677 |
| silent sites,  without recombinants | O_3+4+7_ | -0.448 | -0.312 |

* P-value < 0.05

**Table S6:** Synonymous and nonsynonymous polymorphism and divergence (Ps, Pn, Ds, Dn respectively) and DoS calculated as Dn/(Dn+Ds) - Pn/(Pn+Ps) for O_3+4+1_ and O_3+4+7_ chromosomal arrangements. Divergence was calculated using *D. pseudoobscura* as outgroup. Shadowed genes are located inside inversions.

|  |  | **Ps** | **Pn** | **Ds** | **Dn** | **DoS** |
| --- | --- | --- | --- | --- | --- | --- |
| *Pif1A* | O_3+4+1_ | 0 | 1 | 15 | 0 | -1.000 |
| *Pif1A* | O_3+4+7_ | 0 | 2 | 15 | 0 | -1.000 |
| *Abi* | O_3+4+7_ | 16 | 0 | 54 | 0 | 0.000 |
| *Sqd* | O_3+4+1_ | 1 | 0 | 23 | 4 | 0.148 |
| *Sqd* | O_3+4+7_ | 4 | 0 | 23 | 4 | 0.148 |
| *Yrt* | O_3+4+1_ | 24 | 3 | 57 | 9 | 0.025 |
| *Yrt* | O_3+4+7_ | 28 | 1 | 57 | 9 | 0.102 |
| *Atpα* | O_3+4+1_ | 2 | 0 | 51 | 10 | 0.164 |
| *Atpα* | O_3+4+7_ | 8 | 10 | 47 | 6 | -0.442 |
| *Fmr1* | O_3+4+1_ | 10 | 2 | 37 | 1 | -0.140 |
| *Fmr1* | O_3+4+7_ | 7 | 1 | 37 | 1 | -0.099 |

**Table S7:** Synonymous site and branch-site tests implemented in CodeML of the PAML v4 package^20^. Site tests were performed comparing the neutral model M1a (model=0; NSsites=1) with alternative M2a (model=0; NSsites=2), and the neutral model M7 (model=0; NSsites=7, ncatG=10) with the alternative M8 (model=0; NSsites=8, ncatG=10). For the branch-site test 2, in the neutral model we used the parameters model=2; NSsites=2; fix_omega = 1; omega=1 and, for the alternative, model=2; NSsites=2; fix_omega = 0; omega=1.5.

| **Site test** |  |  |  |
| --- | --- | --- | --- |
| **model** | **aa in position 109** | **LRT** | **pval** |
| M1a vs M2a | A | 0 | 1 |
| M7 vs M8 | A | -0.001 | 1 |
| M1a vs M2a | G | 0.125 | 0.939 |
| M7 vs M8 | G | 0.216 | 0.897 |
| **branch-site test 2** |  |  |  |
| **branch** | **aa in position 109** | **LRT** | **pval** |
| O_3+4+7_ | A | 11.428 | 0.0007 |
| O_3+4+7_ | G | 11.773 | 0.0006 |
| O_3+4+1_ | A | 0.426 | 0.514 |
| O_3+4_ | S | 1.20e-05 | 0.997 |
